# A Method for Multimodal Information Extraction and Knowledge Graph Construction in Substation Secondary System

**DOI:** 10.3390/e28060655

**Published:** 2026-06-09

**Authors:** Wenting Zha, Yue Liu, Dengrui Peng, Zhipeng Su

**Affiliations:** 1 School of Mechanical and Electrical Engineering, China University of Mining and Technology-Beijing, Beijing 100083, China; wtzha@cumtb.edu.cn (W.Z.); zqt2500403109@student.cumtb.edu.cn (D.P.); 2 State Grid Anhui Electric Power Co., Ltd., Wuhu Power Supply Company, Wuhu 241000, China; 13966004882@sohu.com

**Keywords:** substation secondary system, information extraction, multi-source information alignment, knowledge graph

## Abstract

Multi-source heterogeneous data in substation secondary systems are typically characterized by high entropy and disorder, which pose significant challenges for cross-modal information integration and efficient retrieval. Therefore, a method for multimodal information extraction and knowledge graph construction is proposed, enabling structured processing of heterogeneous data from multiple sources. For the image modality, positional and semantic information is extracted using YOLOv8n and Optical Character Recognition (OCR) techniques. To mitigate the effects of uncertain connection topology and noise interference, a Heuristic Circular Stepping Search Algorithm (HCSA) is designed to achieve deterministic path tracing of information flows. For the text modality, a RoFormer-BiLSTM-CRF model enhanced with Rotary Position Embedding (RoPE) is developed to alleviate information degradation in long-sequence texts, thereby enabling high-accuracy extraction of entities and relationships. Furthermore, by combining the domain ontology mapping rules and string similarity, the extracted device entities from the two modalities are aligned, thereby converting scattered data into a structured knowledge graph. Experiments conducted on the secondary-side data of a substation in China demonstrate that the proposed method effectively extracts multimodal information from substation secondary systems, providing valuable support for information management and decision-making assistance in complex industrial systems.

## 1. Introduction

With the continuous advancement of smart grid development, substation secondary systems have accumulated massive volumes of operation and maintenance data from multiple heterogeneous sources, as they play a critical role in ensuring the secure and stable operation of power grids. Such data are often stored in a scattered manner in the form of isolated single-page diagrams or unstructured text, exhibiting typical characteristics of high entropy and disorder. In complex operational scenarios, such as signal topology tracing across different panels and collaborative maintenance across different bays, the weak correlation among heterogeneous data features results in cumbersome workflows and low efficiency in information retrieval and logical association. Knowledge graphs provide strong capabilities in semantic association, reasoning, and information integration. By establishing deterministic association mappings among heterogeneous nodes from multiple sources, fragmented and discrete data can be reconstructed into a highly structured relational network. This offers theoretical support for the structured representation and intelligent retrieval of information in the power domain [1].

Extensive research has been conducted globally on the reconstruction of unstructured data in the power domain into structured knowledge graphs. For the text modality, Natural Language Processing (NLP) technology is widely used to extract entity and relation information from unstructured text sequences [2]. Among these, methods based on pre-trained language models and their hybrid architectures have become the dominant paradigm for information extraction in this field, owing to their superior capability in capturing global semantic distributions [3,4,5]. Ther authors of [6] employed the Bert-BiLSTM-CRF model to extract entities and relations associated with power equipment. By integrating traditional keyword filtering with subgraph querying techniques, this approach effectively enhanced both the query accuracy and response speed for technical standards and product information concerning power equipment. Ref. [7] utilized the Bert-BiLSTM-ATT model to extract diverse entities and relations, including devices, credentials, timeframes, and locations. This methodology facilitated the construction of corpus resources for power service centers and a specialized knowledge base for the power sector. In terms of textual feature fusion and alignment, ref. [8] proposed an enhanced ERNIE-CNN model that incorporates an attention mechanism to effectively integrate semantic features and spatial patterns within operational texts. Ref. [9] proposed a learnable convolutional attention network for unsupervised entity alignment that effectively captures structural information while reducing the overlap of redundant information, thereby providing valuable insights for entity alignment in the power domain. Ref. [10] introduced a model architecture for attribute type recognition based on knowledge graphs. By transferring the inferred fine-grained type probability distribution of target objects and integrating it with the outputs of conventional generative models, the model predicts the distribution over the complete set of types. These techniques have achieved remarkable performance in applications such as power equipment information retrieval, fault diagnosis, and decision support [11,12,13]. However, entities in substation secondary systems are often characterized by diverse forms, ambiguous boundaries, and long-range dependencies, which still limit the accuracy of entity and relation extraction achieved by existing methods.

Regarding the image modality, computer vision techniques have been widely applied to tasks such as appearance recognition of substation equipment, automatic meter reading acquisition, and switchgear status perception [14,15]. Ref. [16] employed D-LLE manifold learning and the Canny algorithm for local dimensionality reduction and edge detection of graphical features. By integrating YOLO-based object detection with OCR-based text recognition, semantic reconstruction of graphical nodes and connection relationships in secondary wiring diagrams was achieved. Based on ontology modeling theory, ref. [17] extracted multidimensional entities and their attribute topologies from unstructured high-dimensional visual streams such as equipment photographs and surveillance videos, thereby providing effective support for the evolution of low-level observational data into structured knowledge graphs and their dynamic adaptive updating. The authors of [18] modified image features, including positional information and edge connectivity, through self-supervised learning during the pre-training stage. Combined with transfer learning and category semantic fusion modules, this approach enabled automatic detection of conventional defects in transmission lines, thereby reducing the risks and computational costs associated with manual inspection. In addition, the authors of [19] abstracted physical grid topology, equipment metadata, and electrical connection states into multidimensional nodes and relational edges in graph space. Based on a breadth-first multi-source path search algorithm, automated topological tracing of electrical paths and hierarchical upstream–downstream division were realized. However, existing studies mainly focus on object detection based on equipment appearance or simple operational states, while research on deep topological logic analysis of complex engineering drawings remains relatively limited.

As the information dimensions of complex systems continue to expand, information networks constructed from a single modality are no longer sufficient to comprehensively represent the complex physical and logical states of power systems. Multimodal information fusion techniques enable the integration of fragmented local information into a globally consistent Multimodal Knowledge Graph (MMKG) through cross-dimensional semantic alignment and feature complementarity [20]. Ref. [21] proposed a Semantic Enhanced Cross-modal Collaborative Attention Network (SCCN) that employs a collaborative attention mechanism to achieve effective cross-modal fusion between textual and visual information. Ref. [22] proposed an information extraction and retrieval-augmented generation (GAT-RAG) method based on graph attention (GAT) networks. This methodology constructs a unified knowledge graph that integrates textual, visual, and structural data. Additionally, it utilizes graph neural networks to rigorously model the semantic dependencies and contextual relations among entities. Ref. [23] realized the high-precision semantic alignment and feature fusion of power equipment images and text entities by introducing a feature extraction network based on Vision Transformer and combining momentum contrastive learning and cross-modal attention fusion mechanisms, which significantly improved the retrieval performance of the multimodal power knowledge graph. Ref. [24] utilized natural language processing and image recognition technologies to analyze the alarm signals of substation equipment, as well as the forms and parameters of the equipment. Eventually, it integrated multi-source data to achieve intelligent handling strategies for substation alarms, providing a technical foundation for the monitoring, processing, and decision-making services of inspectors.

To address the inefficiencies in information retrieval and correlation matching within complex operation and maintenance scenarios of substation secondary systems, on the basis of deep analysis of the characteristics of multimodal data on the substation secondary side, this paper defines an ontology model oriented to this field, formulates standardized triple mapping rules, and further proposes an information extraction and knowledge graph construction framework for information flow diagrams and safety-measure tickets. The main contributions of this paper are summarized as follows:

- Aiming at the problem of the difficulty of topology parsing caused by dense graphic elements and messy intersecting lines in the information flow diagram, the Heuristic Circular Stepping Search Algorithm (HCSA) is innovatively designed. By incorporating a dynamic directional masking strategy and an extremum-point identification mechanism, the algorithm effectively suppresses local topological noise, enabling deterministic reconstruction of information flow paths and accurate extraction of entity connectivity relationships in complex directed networks.
- To address the issues of contextual information attenuation and high entity boundary uncertainty in long-sequence, unstructured instructions, a RoFormer-BiLSTM-CRF hybrid information extraction model enhanced with rotary position embedding in the underlying layer is constructed. By transforming absolute positional encoding into relative distance-aware representations between characters, the model effectively mitigates semantic information loss caused by long-range dependencies, thereby enabling high-precision extraction of entities and relationships from textual data.
- To overcome the limitations of information expression in a unimodal data source, cross-modal entity matching of image and text information is conducted based on string similarity to construct an MMKG that encompasses key elements such as equipment, information flow directions, and circuit wiring terminals. This provides a valuable reference for the intelligent management and control of power systems, as well as the efficient retrieval of multimodal information.

## 2. Framework for Knowledge Graph Construction

This chapter analyzes the characteristics of multimodal data and defines the domain ontology model of the secondary side of the power system. On this basis, a comprehensive framework for constructing a multimodal knowledge graph is proposed.

### 2.1. Multimodal Data Feature Analysis

Multimodal data on the secondary side of the substation include cable information flow diagrams, site survey forms, secondary operation safety-measure tickets, protection replacement drawings, etc. This paper mainly focuses on information flow diagrams and safety-measure tickets as the data basis for research.

**Figure 1:**
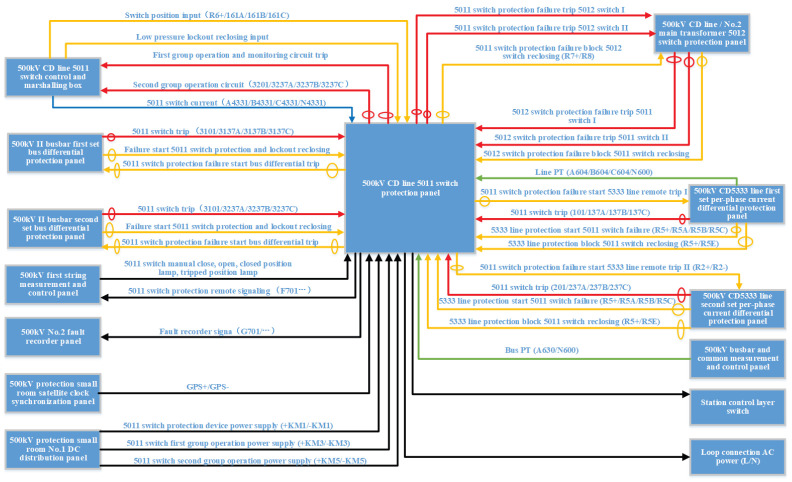
Example information flow diagram.

As representative data within the image modality, information flow diagrams primarily delineate the physical and spatial interconnections and signal transmission logic among various devices. The blue rectangular boxes in Figure 1 identify secondary-side substation cabinets or protection devices, and the multi-colored directed arrows and the text above them characterize the connection relationships and signal flows between the main equipment and associated equipment, which have the characteristics of complex spatial topology and severe overlapping and coupling of graphics and text.

However, information flow diagrams are usually stored in a scattered manner in the form of isolated single-page drawings, which can only present the physical topology of local subsystems or a single piece of main equipment and cannot intuitively reflect the global interconnection status of the secondary-side power-system equipment in its entirety. In scenarios such as information flow tracing across different panels and cabinets or complex fault troubleshooting, maintenance personnel need to carry out cumbersome manual browsing and logical integration among a large number of drawings, which makes the retrieval of equipment associated across different drawings extremely difficult. Therefore, it is imperative to perform structured analysis of the information flow diagrams for the entire station, transforming the physical associations in discrete drawings into deterministic graph data structures, effectively eliminating the topological uncertainty of local representations and thereby achieving accurate retrieval of equipment associated across drawings and tracing of signal flow throughout the entire chain.

Secondary safety-measure tickets refer to textual data that are pre-compiled, audited, and executed on site during the maintenance, testing, or modification of secondary equipment in operational power systems (such as relay protection devices, automatic devices, and monitoring and control devices) to achieve physical or logical isolation between “maintenance equipment” and “operational equipment” in electrical circuits and logical pathways. Safety-measure tickets are usually stored in a scattered manner in the form of unstructured, independent text. The traditional mode, which relies on operation and maintenance personnel to manually read scattered drawings line by line based on experience and perform cross-checking, is inefficient and highly susceptible to logical oversights and potential misoperation risks stemming from human fatigue. Table 1 shows some examples of safety-measure entries that contain diverse forms of equipment and terminal entities. The position and boundary information of these entities is uncertain, and there are long-range dependencies between entities, which are extremely sensitive to the relative positions between characters. Therefore, it is necessary to extract granular information regarding equipment, terminals, and actions from each operation record and perform cross-modal information integration and global mapping with the topological relationships between devices in the information flow diagrams so as to advance the intelligent operation and maintenance capabilities of substation secondary systems.

### 2.2. Construction of the Domain Ontology for Substation Secondary Systems

Ontology serves as the schema layer and data skeleton of the knowledge graph, defining the conceptual hierarchies, entity types, and permissible relational rules within a specific domain [25]. In order to eliminate the heterogeneous data gap between information flow diagrams and safety-measure tickets while accurately representing the topological connections and terminal-side equipment operation semantics of the secondary system, a substation secondary-side domain ontology model is constructed in a top-down manner. This model is established on the basis of an in-depth analysis of the heterogeneous characteristics of secondary-side multimodal data and combines power-domain expert knowledge with business-logic deconstruction, which defines three primary categories of core entities and three fundamental types of semantic relationships. As shown in Table 2, in the entity dimension, this paper defines “Equipment” as the entity representing all secondary devices, the “Loop” as the entity representing circuit information, and the “Terminal” entity as the business execution unit; in the relationship dimension, it defines the “Information Flow” as the relationship representing physical cable routing, the “Action” as the relationship depicting business operation logic, and “Subordination” as the relationship mapping different logic circuits and equipment terminal levels.

Based on the standardized domain ontology model mentioned above, this study formulates specialized triple mapping mechanisms for both image and text modalities. Within the image modality, the model extracts <Equipment, Information Flow, Equipment> triplets to convert unstructured imagery into a directed topological network, thereby reconstructing the fundamental physical backbone of the substation secondary system. Regarding the text modality, the model extracts <Equipment, Action, Loop> and <Loop, Subordination, Terminal> triples, defining the business operation scope of operation and maintenance instructions and accurately restoring the hierarchical subordinate structure from loop to terminal inside the equipment. These mapping mechanisms transform heterogeneous multimodal data into a structured representation that intertwines physical topology with operational logic, effectively making up for the semantic deficiency of a single modality in information representation. Furthermore, this approach establishes a standardized schema-layer foundation, which is essential for the subsequent development of information extraction models and the alignment of multi-source entities.

### 2.3. The Framework for Constructing Knowledge Graphs Based on Multimodal Data

**Figure 2:**
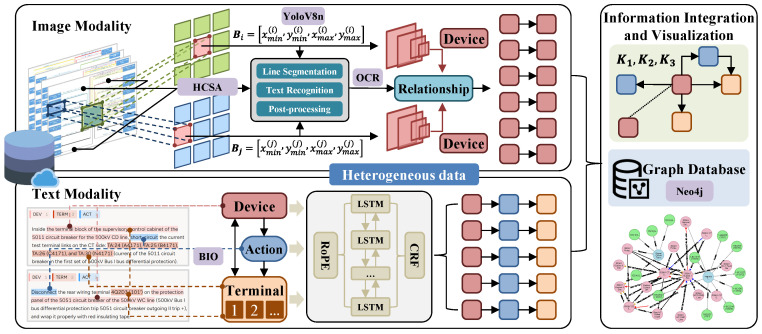
Overall framework for knowledge graph construction based on multimodal data.

Based on the ontology model of substation secondary systems and the mapping rules of triplets among different modalities, this paper proposes a general framework for constructing a multimodal knowledge graph oriented towards information flow diagrams and safety-measure tickets. As shown in Figure 2, the framework is mainly composed of three core modules working together. Within the image modality, an information extraction model for information flow diagrams is constructed. This model integrates YOLOv8n, OCR, and spatial topological analysis technology based on the HCSA. It accurately locates the device nodes from unstructured pixel drawings and tracks the topology link directions, thereby extracting the information flow relationships between devices and constructing the physical topology framework of the secondary-side knowledge graph of the substation. Regarding the text modality, an information extraction model for safety-measure tickets based on RoFormer-BiLSTM-CRF is constructed. By integrating a pre-trained language model with deep learning networks, the model accurately identifies equipment, terminal, and operational entities within complex and unstructured long-form text, thereby filling the terminal-level dynamic business logic into the equipment-level physical topology skeleton. Based on this, cosine similarity is used to perform synonymous disambiguation for heterogeneous elements and to align entities for cross-modal triples. Finally, the aligned text and image triples are stored and visualized in the Neo4j graph database, constructing a multimodal knowledgetions of information expression in substation.

## 3. Multimodal Information Extraction and Alignment

Based on the constructed knowledge graph framework, this chapter introduces information extraction methodologies for both image and text modalities and performs alignment of information from different modalities to achieve the construction of triples in the multimodal knowledge graph.

### 3.1. Information Extraction for the Image Modality

To address the limitations of traditional single-image processing algorithms in capturing deep semantic relationships within information flow diagrams, this part introduces an information extraction methodology that integrates object detection, OCR, and the HCSA, realizing extraction from disordered pixels to structured graph triplets.

#### 3.1.1. Entity Extraction Method of Information Flow Diagrams

Equipment entities within substation secondary system schematics are visually characterized by blue rectangular frames. The YOLOv8n model is used for entity localization, and it outputs a sequence of target bounding-box coordinates, along with their corresponding confidence scores. Given an input image with spatial dimensions of width *W* and height *H*, the predicted coordinates for the *i*-th entity bounding box are denoted as $B_{i}=\left[x_{\min}^{\left(i\right)},y_{\min}^{\left(i\right)},x_{\max}^{\left(i\right)},y_{\max}^{\left(i\right)}\right].$ In order to eliminate the influence of the image-resolution scale on the subsequent spatial topological calculations, this paper performs global normalization on the predicted bounding-box coordinates and calculates the geometric center-point coordinates of each equipment entity as follows:

(1) $$c_{x}^{\left(i\right)}=\frac{x_{\min}^{\left(i\right)}+x_{\max}^{\left(i\right)}}{2W},\;c_{y}^{\left(i\right)}=\frac{y_{\min}^{\left(i\right)}+y_{\max}^{\left(i\right)}}{2H}$$

The normalized geometric centroids exhibit scale-invariant properties within the topological space, which provides a unified spatial metric baseline for subsequent Euclidean distance measurements and topological link anchoring across diverse equipment nodes. Based on this, considering that the information flow diagram has a high level of clarity and the text contained therein is in regular fonts, without any issues such as irregular fonts or blurred characters, this paper uses PaddleOCR 3.1 for text recognition and conducts error detection and verification through manual inspection methods. Eventually, the text labels of the entity nodes in the image can be obtained, achieving the cross-modal transformation of device entities from visual features to semantic entities.

#### 3.1.2. Relationship Extraction Method of Information Flow Diagrams

The information flow diagram represents the topological connection relationships and signal flow between devices using directed arrows of different colors. However, there is a large number of dense attribute texts and overlapping graphic elements in the diagram, which causes serious interference in the identification of the topological relationships between devices. Therefore, in this paper, HSV (Hue, Saturation, Value) color-space technology is utilized to extract the connection lines between devices from the complex background, generating a binarized mask image. Subsequently, morphological erosion is applied to eliminate isolated pixel-level artifacts and textual edge adhesions, finally extracting the connection topology skeleton.

However, accurately determining arrow orientations within a connection topology is the critical challenge in extracting inter-device relationships. It is difficult to achieve path tracing and endpoint recognition with arrow connections using traditional image processing algorithms. Specifically, when the Zhang–Suen thinning algorithm processes hollow ellipses superimposed on line segments and small triangles at arrow tips, it generates closed-loop skeletons and topological spurs. This leads to the extraction of numerous pseudo-endpoints, making it impossible to uniquely determine the true endpoints of the connection topology. The Pavlidis algorithm a contour-tracing method based on local pixel neighborhoods, is primarily suitable for extracting the closed boundaries of standard connected regions. Lacking the capacity to extract global geometric features, this method cannot directly locate and output the terminal coordinates of a connection line. While the Breadth-First Search (BFS) algorithm performs well in identifying the endpoints of simple lines, its accuracy decreases significantly in the presence of intersecting lines or overlapping hollow ellipses [26]. Although the Hough transform is effective for extracting global straight-line segments, applying it to detect polylines in information flow diagrams yields numerous isolated horizontal and vertical segments, thereby failing to directly output the true endpoints of the connection lines.

Therefore, this paper innovatively proposes the HCSA. By establishing a detection circle based on the geometric centroid for iterative stepping, the algorithm strictly constrains the forward trajectory. Furthermore, by incorporating direction masking, extreme point detection, and branch detection mechanisms, it effectively overcomes interference from local noise and complex edges. This ensures continuous and stable path tracing, alongside the precise localization of true endpoints, even in the presence of multiple angular turns along the lines. The procedural framework is shown in Algorithm 1.

Specifically, a circular detection window with a radius of *R* is first constructed, using the geometric centroid ($p_{c}$) of the connection contour as the initial reference point. The step radius (*R*) is subject to the geometric constraint of $D_{\max}^{\mathrm{line}\_\mathrm{wide}}<R\leq D_{\min}^{\mathrm{line}}$, where $D_{\max}^{\mathrm{line}\_\mathrm{wide}}$ represents the maximum pixel width of the lines within the mask image and $D_{\min}^{\mathrm{line}}$ represents the minimum pixel length of the line segment on the fold line. Based on the spatial intersections between the detection circumference and the binary mask of the connection lines ($M_{c}$), the algorithm detects the local extension directions of the link at the initial position and adds these directions to the search queue as active paths. Subsequently, using the current endpoint of the active path ($p_{\mathrm{curr}}$) as the center, the algorithm performs multi-step iterative stepping along the connection trajectory. The maximum number of steps is defined as $S_{\max}=L_{\max}^{\mathrm{line}}/R$, where $L_{\max}^{\mathrm{line}}$ signifies the maximum pixel length of the line.
**Algorithm 1:** Heuristic Circular Stepping Search Algorithm (HCSA).
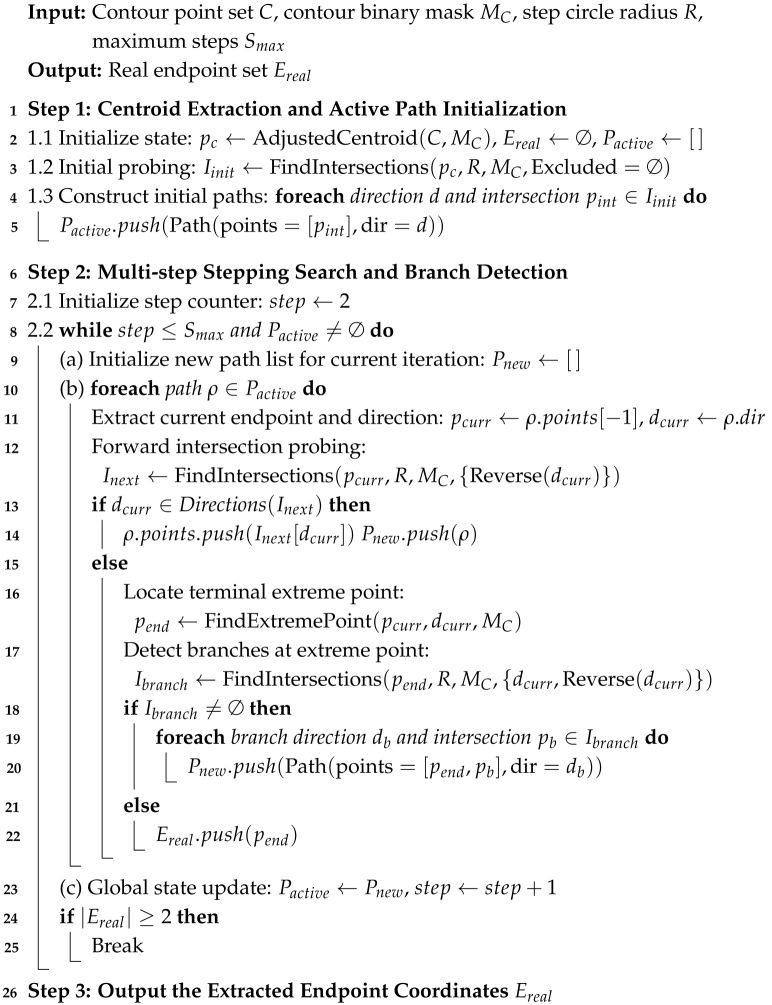


To mitigate the interference caused by circular overlapping graphical primitives during the stepping progression, the algorithm introduces a dynamic direction-masking strategy before each search for forward intersections. Upon the successful detection of a new valid intersection along the current traversal heading, this point is designated as the subsequent stepping benchmark to facilitate unidirectional path extension. If the circular detection window fails to identify valid intersections along the specified trajectory, indicating that the path has either encountered a geometric corner or reached its topological termination, the algorithm is programmed to pinpoint the local extreme point ($p_{\mathrm{end}}$) in the current direction. Subsequently, exploratory branching detection is executed at this location. Should a novel branch deviating from the original traversal heading be detected, a new search path is instantiated. Conversely, if no such branch exists, the point is classified as a genuine topological endpoint and integrated into the endpoint repository ($E_{\mathrm{real}}$). By transforming continuous pixel traversal into discrete directed stepping, the HCSA incurs a computational cost of only O(R) for scanning a circumference of radius *R* per step. Combined with the maximum number of tracking steps ($S_{\max}$), the theoretical time complexity of the algorithm for extracting a single connection line is $O\left(S_{\max}\times R\right)=O\left(L_{\max}^{\mathrm{line}}\right)$, exhibiting a linear relationship with the length of the target line.

After successfully extracting the coordinates of the two endpoints of the connection line, it is necessary to distinguish between the tip and the tail of the arrow. By analyzing geometric features, it can be seen that the arrow tip is usually a small triangle, while the arrow tail transitions smoothly with the main line, meaning the tip has more local white pixel blocks than the tail. Therefore, an endpoint polarity discrimination function based on local pixel density is constructed. By comparing the pixel density features of the two endpoints, the framework precisely identifies the specific flow of signal transmission and achieves logical transformation from an undirected topology to a directed relationship graph.

#### 3.1.3. Generation of Information Flow Triples

After identifying the tips and tails of the connection lines, Euclidean distance measurement is utilized to match the equipment connection relationships. Assuming that *N* equipment entities are detected in the image, the set of their globally normalized center-point coordinates is denoted as $C=\{c_{1},c_{2},\ldots ,c_{N}\}$. For any given extracted, directed link ($L_{k}$) with a known tip ($p_{k}^{\mathrm{tgt}}$) and tail ($p_{k}^{\mathrm{src}}$), by minimizing the Euclidean spatial distance, the two ends of the link are precisely anchored to the corresponding sending equipment and receiving equipment.

To endow topological connections with specific business semantics, it is necessary to accurately map the attribute texts scattered around the links to the corresponding directed edges. Using the Hough line detection algorithm to extract the ordinate ($y_{k}$) of the main line of the link and combining the horizontal boundary domain ($\left[x_{k}^{\min},x_{k}^{\max}\right]$) of the link, a label recognition region ($\Omega_{k}$) is adaptively constructed for each directed edge. Assuming the center point of the *j*-th text bounding box obtained by OCR is $t_{j}=\left(x_{j},y_{j}\right)$, the discrimination criterion for its spatial subordination relationship with a specific link is defined as follows:

(2) $$t_{j}\in \Omega_{k}\Leftrightarrow \left\{\begin{array}{c} x_{k}^{\min}\leq x_{j}\leq x_{k}^{\max}\\ y_{k}\leq y_{j}\leq y_{k+1} \end{array}\right.$$

If the text center point strictly satisfies the above geometric inclusion conditions, it is determined that the text belongs to this connection line, realizing the transformation of unstructured text into semantic link attributes. Through the spatial mapping and logical aggregation of the source device name, target device name, signal transmission orientation, and corresponding attribute texts, the raw image pixels and character elements within the schematic are effectively converted into a structured knowledge graph triplet set ($K_{1}$), which is defined as follows:

(3) $$K_{1}=\left\{\langle e_{dev\_src},e_{line},e_{dev\_tgt}\rangle |e_{dev\_src},e_{dev\_tgt}\in E_{dev},e_{line}\in E_{edge}\right\}$$

where $e_{dev\_src}$ and $e_{dev\_tgt}$ respectively represent the source equipment and target equipment of the topological link, $e_{line}$ is the connection relationship between pieces of equipment, $E_{dev}$ is the equipment entity set extracted by detection, and $E_{edge}$ is the complex semantic edge set that fuses signal attributes and directional connection relationships.

### 3.2. Information Extraction for the Text Modality

To address the challenges of heterogeneous text formats and complex entity extraction in safety-measure tickets, a RoFormer-BiLSTM-CRF based information extraction model is developed, enabling accurate extraction of textual triples.

#### 3.2.1. Rotary Position Embedding

**Figure 3:**
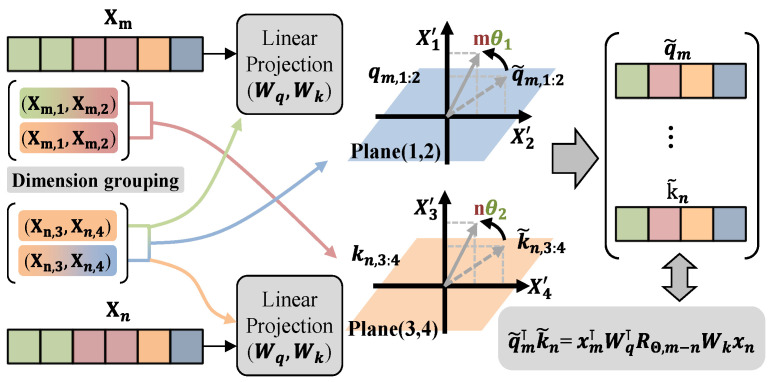
Schematic diagram of rotation position encoding.

Traditional pre-trained models utilizing absolute position embedding, such as Bert, are highly susceptible to feature extraction degradation and positional information decay when processing texts characterized by long-range inter-entity dependencies and intra-entity character position sensitivity. Therefore, this paper introduces the Rotary Position Embedding mechanism [27] in the bottom-layer feature extraction stage. It realizes position encoding by mapping the context representation to the complex space and multiplying it by an orthogonal rotation matrix determined by the absolute position. When calculating the inner product of the self-attention mechanism, this mechanism can mathematically equivalently transform the absolute position index into the relative distance perception between characters; its principle logical architecture is shown in Figure 3.

Specifically, let the input textual sequence of the safety-measure ticket be denoted as $X=\{x_{1},x_{2},\ldots ,x_{L}\}$. Suppose the word-embedding vectors at positions *m* and *n* are linearly projected to yield the *d*-dimensional query vector ($q_{m}$) and key vector ($k_{n}$), respectively. The RoPE mechanism, by constructing orthogonal rotation matrices $R_{\Theta ,m}$ and $R_{\Theta ,n}$, explicitly injects the absolute position information into the corresponding vectors in the form of a rotation operation. After fusing the position information, vectors ${\tilde{q}}_{m}$ and ${\tilde{k}}_{n}$ can be formulated as follows:

(4) $${\tilde{q}}_{m}=R_{\Theta ,m}W_{q}x_{m}$$

(5) $${\tilde{k}}_{n}=R_{\Theta ,n}W_{k}x_{n},$$

wherein the rotation matrix ($R_{\Theta ,m}$) possesses orthogonality. Based on the geometric properties of rotation matrices within Euclidean space, it follows that $R_{\Theta ,m}^{⊤}R_{\Theta ,n}=R_{\Theta ,n-m}$. Therefore, when calculating the inner product of self-attention, the product of the absolute position matrices can achieve elimination, making the inner product of the two vectors equivalently transformed into a function depending only on the relative position (m−n):

(6) $${\tilde{q}}_{m}^{⊤}{\tilde{k}}_{n}={\left(R_{\Theta ,m}W_{q}x_{m}\right)}^{⊤}\left(R_{\Theta ,n}W_{k}x_{n}\right)=x_{m}^{⊤}W_{q}^{⊤}R_{\Theta ,m-n}W_{k}x_{n}$$

As indicated by the preceding equation, RoPE mathematically converts the additive constraints of absolute positions into a rotational operation applied to the feature vectors dictated by their positional indices. This mechanism not only preserves the norm invariance of the word embeddings but also endows the model with an exceptional length extrapolation capability, significantly enhancing the extraction ability of the network for deep semantic features in the complex long instructions of safety-measure tickets [28].

#### 3.2.2. Text Information Extraction Method Based on the RoFormer-BiLSTM-CRF Model

**Figure 4:**
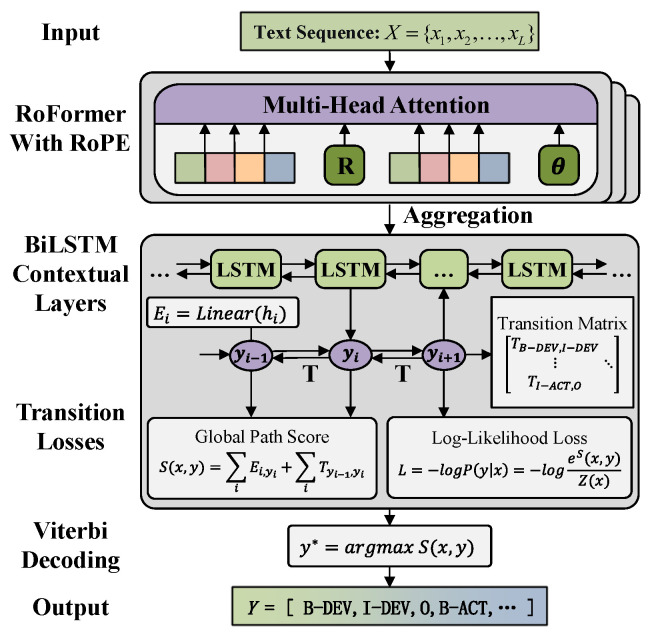
Diagram of the information extraction architecture for the text modality.

To address the ambiguity of entity boundaries and the challenges of long-range dependencies within the lengthy texts of safety-measure tickets, this study constructs a joint information extraction architecture based on RoFormer-BiLSTM-CRF, as illustrated in Figure 4. During the data processing phase, manual sequence annotation is performed on the safety-measure documents utilizing the Label Studio platform. By employing the BIO tagging scheme, three core entity categories are explicitly defined: equipment (DEV), terminal (TERM), and action (ACT). In the feature encoding phase, a sequence of feature vectors infused with relative positional information is acquired via the RoPE mechanism. Assume the feature sequence output by the RoFormer layer is $H=[h_{1},h_{2},\ldots ,h_{n}]$, where *n* is the sequence length of the input text. To further capture the deep temporal and contextual dependencies within the text, the model feeds the sequence (*H*) into a BiLSTM network for bidirectional encoding. By concatenating the forward and backward hidden states at each time step, a comprehensive representation ($u_{t}=\left[\vec{h_{t}};\overset{\leftarrow }{h_{t}}\right]$) encapsulating global contextual features at time step *i* is obtained. To alleviate the overfitting phenomenon under small sample data, a random dropout deactivation operation is applied to $u_{t}$. Subsequently, it is mapped to *k*-dimensional label space through a fully connected layer, generating the emission score ($E_{i}=\mathrm{Linear}\left(h_{i}\right)$). Thus, the emission score matrix is constructed as $E\in R^{n\times k}$, where *k* is the total number of label categories and $E_{i,y_{i}}$ represents the un-normalized score of the *i*-th character being assigned the label of $y_{i}$.

Due to the strict dependency constraints among entity labels in sequence labeling tasks, relying solely on emission scores is highly likely to result in illegal label transitions. Therefore, this paper introduces a Conditional Random Field (CRF) module at the top layer of the network to perform global optimal decoding and loss calculation. Assume the transition matrix is $T\in R^{k\times k}$, where element $T_{y_{i-1},y_{i}}$ represents the score associated with transitioning from state tag $y_{i-1}$ to $y_{i}$. For a specific input sequence (*x*) and a corresponding predicted label sequence ($y=[y_{1},y_{2},\ldots ,y_{n}]$), the global path-scoring function is defined as the summation of the emission scores and the transition scores:

(7) $$S\left(x,y\right)=\sum_{i=1}^{n}E_{i,y_{i}}+\sum_{i=1}^{n}T_{y_{i-1},y_{i}}$$

During the model training phase, parameter optimization aims to maximize the conditional likelihood of the ground-truth label sequence. The negative log likelihood is employed as the training objective, which is equivalent to minimizing the sequence cross-entropy between the predicted distribution and the empirical data distribution. The global loss function is expressed as follows:

(8) $$L=-\log P\left(y|x\right)=-\log\frac{\exp(S(x,y))}{Z(x)}$$

where $Z\left(x\right)=\sum_{y^{\prime }}\exp\left(S\left(x,y^{\prime }\right)\right)$ denotes the partition function, which normalizes over all possible label sequences by summing the exponentiated scores under a given input. Minimizing this loss enables the model to suppress invalid label transitions, reduce uncertainty in the decoding space, and jointly optimize both the feature representations and the transition-matrix parameters.

During the model inference phase, the Viterbi dynamic programming algorithm is employed to decode the optimal label sequence ($y^{*}=\arg\max_{y}S\left(x,y\right)$) that achieves the maximum global score. After parsing it into specific entity vocabularies, the equipment entity set ($E_{dev}$), action relationship set ($E_{act}$), and terminal entity set ($E_{ter}$) in the safety-measure ticket instructions can be obtained. In the actual operations of the equipment in the security system, the terminal operations of the devices within the same circuit often exhibit a high degree of consistency. According to the hierarchical structural features of the text of the safety-measure ticket, this section adopts heuristic rules based on document structure parsing to perform top-to-bottom structural tree matching between specific safety-measure instruction items and their belonging-context hierarchical titles, accurately extracting the loop entity set ($E_{cir}$) corresponding to the equipment entities. Based on the above entity sets, combining the substation secondary-side domain ontology model, the equipment and operation actions identified by the model and the associated circuits parsed by structure are semantically integrated to generate the triplet set ($K_{2}$):

(9) $$K_{2}=\left\{\langle e_{dev},e_{act},e_{cir}\rangle |e_{dev}\in E_{dev},e_{act}\in E_{act},e_{cir}\in E_{cir}\right\}$$

where $e_{dev}$ is the specific equipment entity involved in the operation instruction, $e_{act}$ is the specific action of the operation instruction, and $e_{cir}$ is the wiring terminal of the specific loop where the equipment is located. Subsequently, to accurately restore the subordinate structure inside the equipment and clarify the logical circuit where the specific terminal is located, the inclusion relation triplet set ($K_{3}$) is constructed:

(10) $$K_{3}=\left\{\langle e_{cir},r_{inc},e_{ter}\rangle |e_{cir}\in E_{cir},e_{ter}\in E_{ter},r_{inc}\in R_{inc}\right\}$$

where $e_{ter}$ represents the specific device terminal number and $R_{inc}$ represents the containment relationship defined in the ontology layer. Ultimately, the complete set of structured triplets generated by text-modality extraction is $K_{text}=K_{2}\cup K_{3}$, realizing the structured conversion of the unstructured text of the safety-measure tickets and providing data support for the construction of the multimodal graph of substation secondary systems.

### 3.3. Information Alignment

Considering human errors during the dataset compilation process and mistakes existing in the information extraction process, the same information often exists in different textual forms. To resolve this issue, this paper introduces a character-level N-gram technique combined with term frequency–inverse document frequency (TF-IDF) to map the equipment entities extracted from the image and text modalities into a continuous feature-vector space for semantic disambiguation.

The character-level N-gram method decomposes an entity string into a set of overlapping continuous character substrings of length *n*. In this study, to simultaneously capture the local morphological features of specialized vocabulary in the power domain and retain robust sequence patterns, the N-gram range is configured to extract character sequences of length n∈[2,4], representing bigrams, trigrams, and fourgrams. Subsequently, the TF-IDF algorithm is utilized to calculate the statistical weight of each extracted N-gram. This mechanism effectively penalizes high-frequency and generic character combinations lacking distinctiveness, such as common power-system prefixes, while amplifying the weights of unique and highly discriminative substrings, thereby significantly enhancing the robustness of entity representation against noise.

Let the feature vector of the image-modality entity be $V_{img}$ and the feature vector of the text-modality entity be $V_{text}$. The semantic similarity between the two is measured using cosine distance, and the calculation formula is expressed as follows:

(11) $$\mathrm{Sim}\left(V_{\mathrm{text}},V_{\mathrm{img}}\right)=\frac{V_{\mathrm{text}}\cdot V_{\mathrm{img}}}{∥V_{\mathrm{text}}∥\times ∥V_{\mathrm{img}}∥}$$

A similarity-matching tolerance threshold (τ) is established such that two cross-modal entities are identified as referencing the same physical object if and only if $\mathrm{Sim}(v_{text},v_{img})\geq \tau$, at which point a node-merging operation is executed.

## 4. Experiment

To validate the effectiveness of the method proposed in this paper for the construction of a substation secondary-side knowledge graph oriented towards multimodal data, a conventional substation in a province of China is selected as a case study. By integrating seven information flow diagrams and one safety-measure ticket, the physical-connection topology and terminal-level structures of the secondary equipment are successfully modeled and visualized.

### 4.1. Experimental Process

#### 4.1.1. Process of Image-Modality Information Extraction

For the image modality, the experimental dataset consists of 40 information flow diagrams sourced from diverse substations for training and validation, while 7 information flow diagrams from the specific target substation serve as the test set. First, the YOLOv8n object detection model is used to perform visual feature localization of the blue rectangular boxes in the images and combined with OCR technology to accurately identify and record the equipment names and global coordinate information within the boxes. A total of 61 discrete equipment entities are successfully extracted in this stage.

Subsequently, to parse the topological connections between the devices, HSV color-space conversion technology is utilized to filter the directed arrow connections in the information flow diagram, generating the corresponding mask image. In order to select the optimal detection radius (*R*) and the maximum step count ($S_{\max}$) of the HCSA, the arrows in all images are traversed to calculate the endpoint recognition accuracy corresponding to different radius (*R*) values.

**Figure 5:**
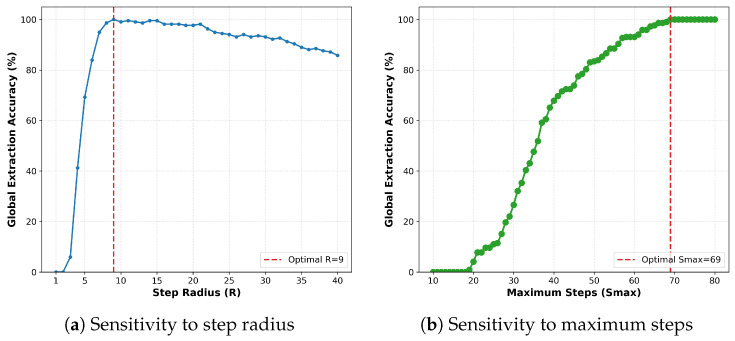
Sensitivity analysis of HCSA parameters.

As shown in Figure 5a, when *R* is relatively small, influenced by the arrows and interference primitives, the endpoint recognition accuracy is relatively low; when R=9, the endpoint recognition accuracy is the highest; and when R>15, the accuracy gradually decreases. This is because the detection radius has already exceeded the pixel length ($D_{\min}^{\mathrm{line}}$) of the smallest line segment on the polyline, causing the terminal part of the polyline to fail to be effectively detected. Therefore, this paper selects R=9 and, based on this radius, further tests the impact of the step count on endpoint recognition. The results are shown in Figure 5b. When the step count exceeds 69, the final endpoint can be stably recognized; therefore, a maximum step count of $S_{\max}=69$ is selected.

**Figure 6:**
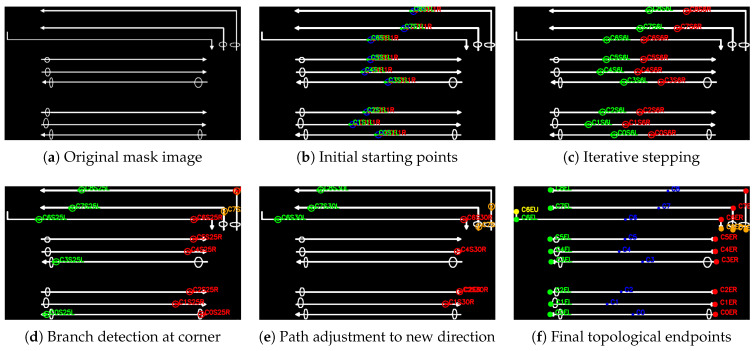
Process diagram of the HCSA.

Utilizing the optimized parameters determined above, the HCSA is applied to automatically trace the paths and identify the endpoints of each directed link within the diagram. Taking a localized region of Figure 1 as an illustrative example, the connection-relationship mask image is presented in Figure 6a, and the intermediate stages of the search process are depicted in Figure 6b–f.

As shown in Figure 6, the geometric centroids of each directed arrow contour are taken as the initial detection starting points, as shown by the blue solid dots in Figure 6b, and the initial link-extension direction is identified by a detection circle with a set radius. As shown in Figure 6c, the algorithm first performs iterative stepping along the identified initial extension directions (red and green paths). When encountering geometric corners, as shown in Figure 6d,e, the intersection points in the original traveling direction disappear, and the algorithm triggers a branch detection mechanism to rescan feasible directions, dynamically adjusting the path from the initial direction to a new direction (such as the orange path). Then, it continues to detect along the new direction; when the detection circle cannot detect valid intersection points in any direction, the algorithm determines that the end of the link has been reached, finally identifying the topological endpoint set as shown in Figure 6f.

Finally, combining morphological shapes and pixel density characteristics, the head and tail of each directed arrow are distinguished based on the white pixel count within a neighborhood of the endpoints. Subsequently, Euclidean distance is employed to perform nearest-neighbor matching between these endpoint coordinates and the geometric centers of the previously localized blue rectangular bounding boxes. This methodology successfully yielded 220 inter-device information flow relationships, effectively completing the construction of the physical topological backbone within the image modality.

#### 4.1.2. Process of Text-Modality Information Extraction

Regarding the text modality, to evaluate the generalization ability of the model in actual substation operation and maintenance scenarios, this experiment uses information from the safety-measure tickets of other substations as the training and validation sets and used information from the safety-measure tickets of the target substation as the test set. Label Studio is used to perform manual sequence labeling on the safety-measure tickets. To alleviate the overfitting problem caused by limited sample data, the dataset needs to be expanded. Considering that relevant text data in the secondary-side domain of the power system contain many professional nouns, data augmentation strategies such as back translation or rewriting easily cause semantic deviation. Therefore, this paper introduces a data augmentation strategy based on same-class random entity replacement before model training. The augment factor is set to 2, and the entity replacement probability is set to 0.6. This strategy effectively expands the semantic diversity of the training corpus while maintaining the original grammatical logic.

In the model training phase, the maximum text sequence length is set to 256, and RoFormer is utilized to to capture underlying features embedded with relative positional information. Parameter optimization is executed via the AdamW optimizer in conjunction with a linear learning-rate warmup strategy. The primary hyperparameter configuration includes Batchsize = 4, Initial Learning Rate = 0.0003, and total Epochs = 50. In the subsequent inference and decoding stage, the optimally converged RoFormer-BiLSTM-CRF model is applied to the test set for entity recognition. Ultimately, the total number of successfully extracted device entities, terminal entities, and action relationships is 257, providing terminal-level information elements for the construction of the multimodal graph.

### 4.2. Experimental Evaluation

#### 4.2.1. Comparative Experiment for Device-Connection Relationship Extraction

To validate the efficacy of the HCSA in extracting the connection relationships between devices, this study conducts comparative experiments against five alternative approaches: the 4-connected BFS algorithm, the 8-connected BFS algorithm, the PCA algorithm, the modified Zhang–Suen skeletonization algorithm, and the modified Pavlidis contour-tracing algorithm. Specifically, the modification strategy for the Zhang–Suen skeletonization algorithm involves designing a customized 3 × 3 hollow convolutional kernel to filter the single-pixel skeleton image. By leveraging the topological feature that a true endpoint possesses only a single adjacent pixel on the skeleton, all candidate endpoints are initially identified. Subsequently, a global Euclidean distance maximization criterion is introduced to extract the pair of points separated by the greatest distance, thereby successfully identifying the two true endpoints of a single target arrow. Meanwhile, the improvement strategy for the Pavlidis contour-tracing algorithm primarily entails searching for the pair of pixels on the contour of the connected region that exhibits the maximum straight-line distance; these pixels are then designated as the two endpoints of the connection line.

**Figure 7:**
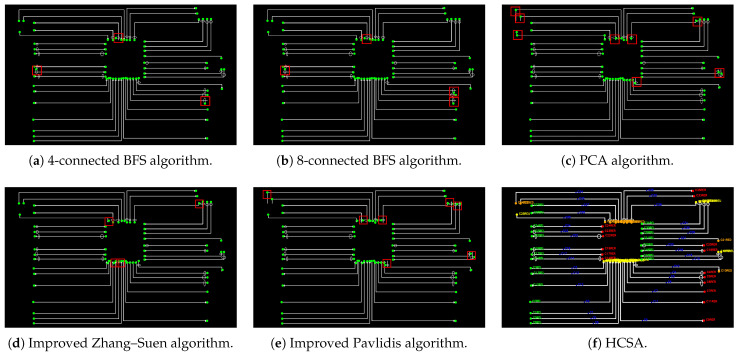
Visual comparison of equipment-connection endpoint identification.

The evaluation utilizes 7 information flow diagrams containing a total of 440 endpoints. Taking the mask image of Figure 1 as an example, the endpoint identification results of each algorithm are shown in Figure 7. The red bounding boxes highlight regions of erroneous identification. It can be observed that the 4-connected and 8-connected BFS algorithms, along with the modified Pavlidis contour-tracing method, are highly susceptible to interference from overlapping closed elliptical elements situated above the arrows, which significantly degrades identification accuracy. When the contour of the arrow has multiple inflection points, the PCA algorithm performs poorly in identifying the endpoints. Influenced by the small triangles at the tips of the arrows, the improved Zhang–Suen skeletonization algorithm easily mistakenly recognizes other triangle vertices at the tips of the arrows as endpoints. Therefore, the HCSA proposed in this paper can effectively overcome the interference of arrow contours, overlapping ellipse elements, and shapes with multiple bends, achieving highly efficient recognition of path tracing and endpoints.

**Figure 8:**
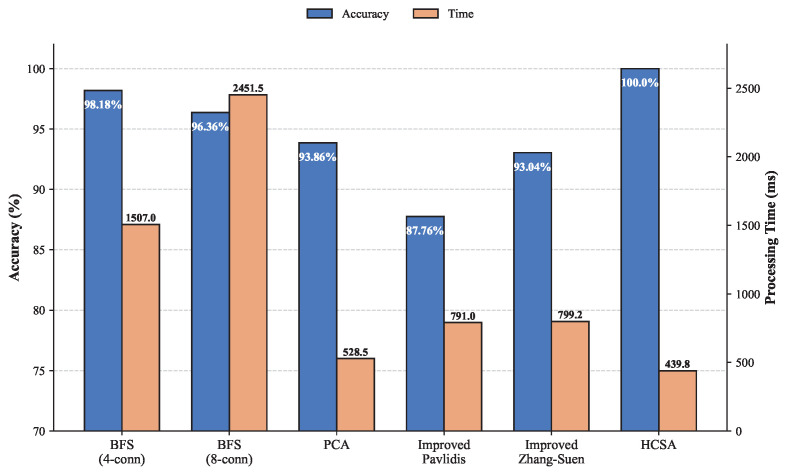
Performance comparison of device-connection relationship extraction.

To quantitatively evaluate the performance advantages of the HCSA, two metrics are analyzed: endpoint detection accuracy ($A=N_{\mathrm{correct}}/N_{\mathrm{total}}$) and processing time. $N_{\mathrm{correct}}$ denotes the number of endpoints correctly detected by the algorithm, and $N_{\mathrm{total}}$ represents the actual total number of endpoints involved in the test. The comparative results are shown in Figure 8, with the primary vertical axis being the accuracy and the secondary vertical axis representing measurement time. As shown in the figure, the HCSA achieves an endpoint detection accuracy of 100%, outperforming the other algorithms. Furthermore, it requires only 439.8 ms of measurement time for endpoint detection and exhibits the lowest algorithmic complexity.

#### 4.2.2. Comparative Experiment on Information Extraction for Safety-Measure Tickets

To validate the effectiveness of the text-modality knowledge extraction model, this paper selects seven schemes, such as RoUIE, GLM-4-FS, Bert-BiLSTM-CRF and Albert-BiLSTM-CRF, as comparative experiments. Five distinct experiments are designed specifically for the GLM-4-FS model. During each iteration, different samples are selected to facilitate learning for the large language model. All other comparative experimental parameters remain identical to those configured for the RoFormer-BiLSTM-CRF model. Repeated experiments are conducted utilizing five different random seeds. Precision (*P*), Recall (*R*), and the F1 score (F1) are adopted as the performance evaluation metrics for all models. The specific calculation formulas are defined as follows:

(12) $$P=\frac{TP}{TP+FP}$$

(13) $$R=\frac{TP}{TP+FN}$$

(14) $$F1=\frac{2PR}{P+R}$$

**Figure 9:**
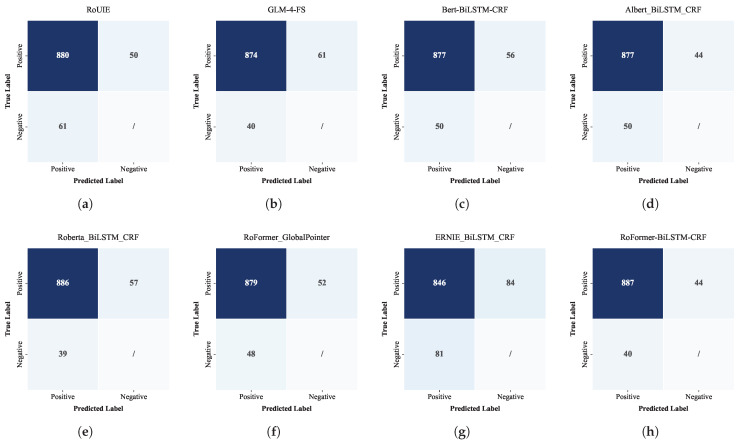
Comparison of confusion matrices of each model.

Specifically, TP denotes the number of entities for which the model correctly identifies both the boundaries and class labels, FP represents the count of entities that are either spuriously extracted or misidentified due to boundary errors, and FN refers to the volume of ground-truth entities that the model fails to detect. Figure 9 presents the confusion-matrix results of each algorithm from a single experimental run. Since GLM-4-FS utilizes cloud-based API calls and possesses inherent generative randomness, the random seed for the remaining comparative experiments is set to 42.

As can be seen from the results, the RoFormer-BiLSTM-CRF model accurately identifies the largest number of positive samples, with a total of TP = 887, which is an increase of 7 compared to the RoUIE model and an increase of 13 compared to GLM-4-FS. Compared with hybrid models such as Bert and ERNIE, the number of false-positive samples is the lowest, which significantly suppresses the misdetection phenomenon. At the same time, its number of missed reported samples is only 40, which is far smaller than other models, and the overall missed reporting level is only slightly inferior to that of the Roberta hybrid model. To verify the stability of the RoFormer-BiLSTM-CRF model, five repeated experiments are conducted. The mean and standard deviation of the evaluation metrics are calculated, and the average inference latency (*T*) per sample is recorded. Specifically, the random seeds for the local models are set to [42, 123, 1024, 2024, 3407]. The evaluation metrics for each model are presented in Table 3.

As presented in the table above, the RoFormer-BiLSTM-CRF model achieves optimal performance across *P*, *R*, and F1 score. These metrics reach 0.9506, 0.9531, and 0.9519 respectively, all demonstrating small standard deviations. Compared to the RoUIE and the GLM-4-FS large language model few-shot fine-tuning approach, the RoFormer-BiLSTM-CRF model improves the F1 score by 0.0096 and 0.0026 respectively. Furthermore, it exhibits less metric fluctuation than GLM-4-FS. The RoFormer-BiLSTM-CRF model achieves an inference latency of 36.14 ms per sample, which is significantly lower than the 302.24 ms required by GLM-4-FS, and remains comparable to other BiLSTM-CRF based models. The RoFormer-GlobalPointer model achieves a lower latency of 26.36 ms, which is attributed to its non-autoregressive matrix-scoring mechanism that eliminates the computational cost of the Viterbi decoding required by the CRF layer. Compared with established hybrid architectures such as Bert, Roberta, and Albert, the RoFormer model leverages RoPE to facilitate the deep integration of relative and absolute positional features, which enables more precise characterization of long-range entity dependencies frequently encountered in safety-measure tickets. Furthermore, regarding the decoding paradigm, the BiLSTM-CRF structure demonstrates higher accuracy than the RoFormer-GlobalPointer model utilizing two-dimensional span extraction with the same backbone. This demonstrates that for documents such as safety-measure tickets that exhibit long-range dependencies and sensitivity to character position, the hard global state-transition constraints provided by the CRF layer can more effectively avoid the generation of illegal label sequences than simple span classification, thereby achieving more precise entity-boundary division.

### 4.3. Ablation Experiment

#### 4.3.1. Ablation Experiments for Image Endpoint Recognition

**Figure 10:**
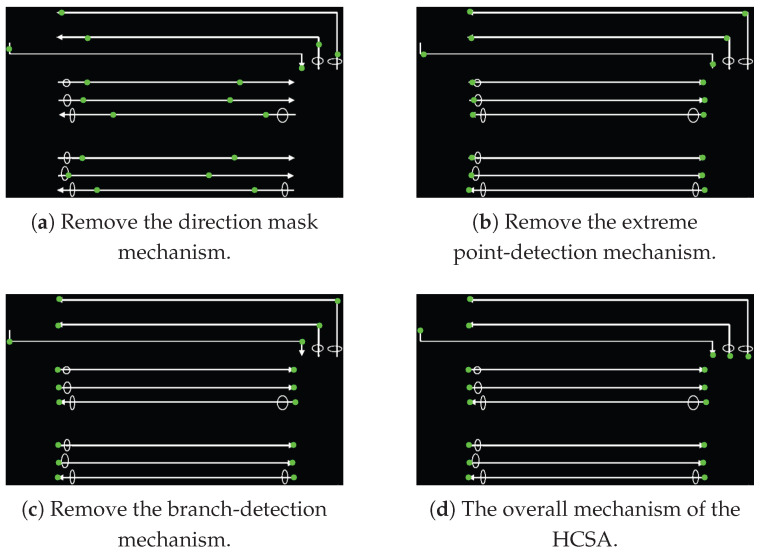
Ablation experiments of each module of the HCSA.

To investigate the impact of individual modules within the HCSA on the endpoint recognition process, this section conducts ablation experiments on all mask images by independently removing the direction-masking, extreme point-detection, and branch-detection mechanisms. Setting R=9 and $S_{\max}=69$ and utilizing selected mask images as representative examples, the final endpoint recognition results are illustrated in the Figure 10. Specifically, Figure 10a demonstrates that when the extreme point-detection mechanism is removed, the search process at each step may track along the previous stepping direction. As depicted in Figure 10b, the absence of the extreme point-detection mechanism also prevents the recognition of inflection points at certain turns, resulting in a failure to detect the subsequent trajectory. Finally, as shown in Figure 10c, eliminating the branch-detection mechanism restricts the search exclusively to the initially identified direction, rendering the algorithm unable to identify alternative stepping directions at turning points.

#### 4.3.2. Ablation Experiments for Text Information Extraction

To compare the performance of the relative position encoding in RoFormer with the absolute position encoding in Bert and to quantify the specific contributions of each module within the RoFormer-BiLSTM-CRF text information extraction model, this section conducts a series of experiments. These experiments independently evaluate Bert, RoFormer, RoFormer combined with BiLSTM, RoFormer combined with CRF, and the complete model. The results are presented in Table 4.

Experimental results indicate that the RoFormer model yields higher precision than the Bert model. However, its lower recall suggests a deficiency in the comprehensiveness of entity recognition, which consequently limits the overall F1 score. When the BiLSTM module is incorporated into this baseline, the model exhibits an enhanced ability to capture bidirectional contextual features over extended distances. This effectively mitigates entity omission in complex contexts, leading to significant improvements in both recall and the overall F1 score while maintaining stable precision. Conversely, if only the CRF module is added to the base model, the precision drops relative to the baseline. Nevertheless, because the CRF layer introduces global boundary constraints by learning label-transition probabilities, it effectively suppresses the generation of invalid label sequences. This mechanism boosts the recall and improves the overall F1 score. Ultimately, the RoFormer-BiLSTM-CRF model achieves optimal performance across the three indicators of precision, recall, and F1 score. It overcomes the precision degradation associated with the use of the CRF module in isolation and successfully circumvents the issue of blurred entity boundaries, which is often induced by absolute position encoding, by effectively capturing contextual dependencies across long distances.

### 4.4. Multimodal Information Alignment and Visualization

**Figure 11:**
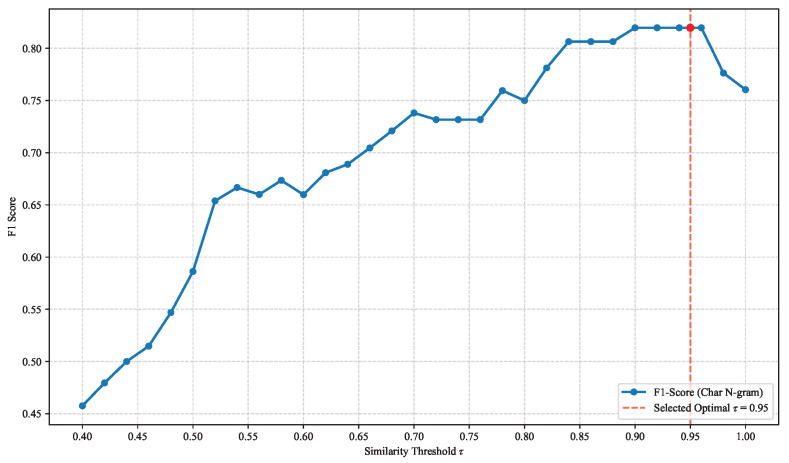
Sensitivity analysis of τ.

To address the inherent noise of the data and potential problems of information extraction inaccuracy, a character N-gram similarity matching strategy is adopted based on the method described in Section 3.3. This maps device entities in the text and image modalities to a unified vector space. A sensitivity analysis is conducted on the similarity threshold (τ) within the range of [0.40, 0.99]. As shown in Figure 11, when 0.9≤τ≤0.96, the F1 score stably remains in the optimal interval. When τ>0.96, the F1 score shows a downward trend. Considering the requirements for the precision of entity alignment in the power domain, this paper selects τ=0.95 as the optimal threshold to align device entities in information flow diagrams and safety-measure tickets.

Combined with the secondary-side domain ontology layer constructed in Section 2.2, the proposed framework ultimately generates a knowledge base comprising 220 $K_{1}$ triplets, 38 $K_{2}$ triplets, and 257 $K_{3}$ triplets. Examples of these triplets are shown in Table 5.

**Figure 12:**
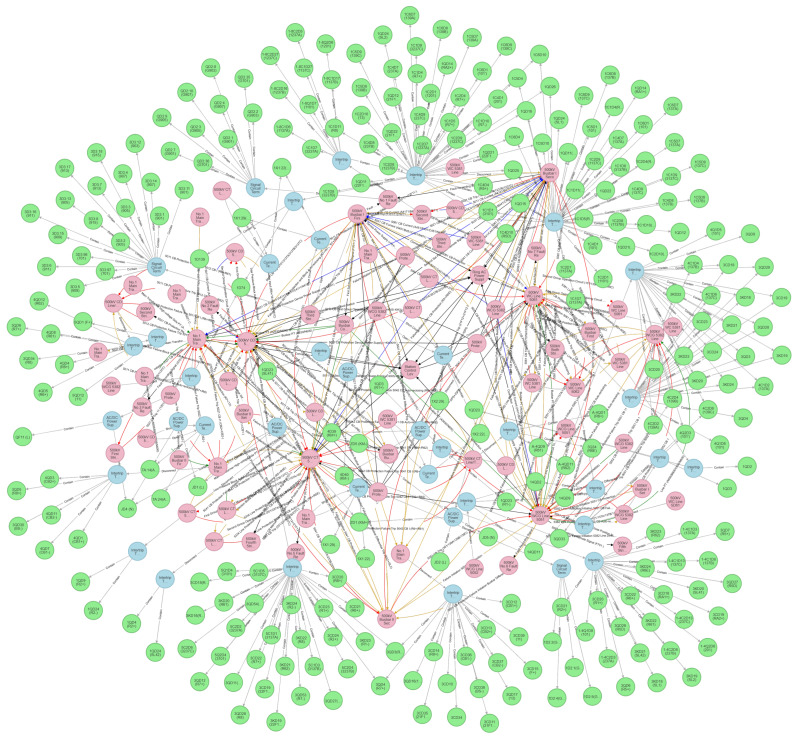
Triplet visualization.

**Figure 13:**
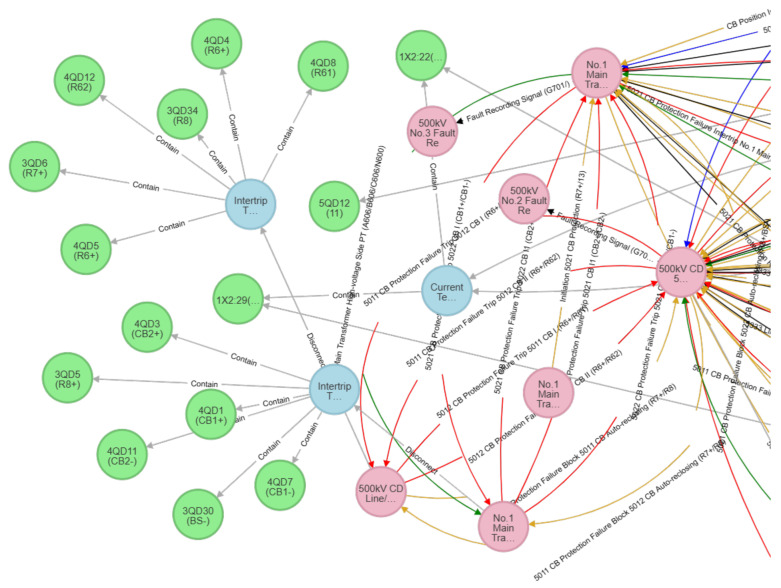
Triplet visualization of local magnification image.

Finally, this study utilizes the Neo4j graph database for a comprehensive visualization of all extracted triplets, as shown in Figure 12, where pink nodes are equipment entities, blue nodes are loop entities, and green nodes are terminal entities. A localized magnification is further provided in Figure 13. The figure shows, in detail, the connection and subordination relationships of equipment, information flow, and terminals, realizing the traceability of information flow between devices and the queryability of different circuit-wiring terminals of the target maintenance equipment. Operation and maintenance personnel can search for target equipment and perform actions on the circuit-wiring terminals according to the graph. For instance, when performing maintenance on the “500 kV CD Line 5012 Switch Protection Panel”, it is necessary to disconnect inter-tripping circuit-wiring terminals such as 4QD4 (R6+) and 4QD8 (R61). Therefore, the knowledge graph construction method proposed in this paper deeply explores the information contained in the information flow graph and the secondary security-measure tickets and converts it into a structured triplet form, achieving the effective integration of image= and text-modality information. This provides an effective reference for solving the problem of diverse and scattered storage of secondary-side data in the power system, which leads to low efficiency in manual information retrieval.

## 5. Discussion

With the development of smart grids, substation secondary systems hae accumulated a massive amount of multi-source heterogeneous data. Traditional multimodal power-system research is mostly limited to fault detection, visual recognition of equipment appearance, or the monitoring of simple operational states of the system and cannot fully solve the problems of cross-drawing signal flow tracing and relational retrieval at the equipment and terminal levels. Therefore, this paper conducted information extraction based on the two modalities of data, including information flow diagrams and safety measure tickets, and ultimately constructed a structured knowledge graph integrating equipment entities, electrical circuits, signal flow directions, and the terminal level.

During the extraction of connection relationships within the image modality, conventional topology extraction algorithms such as BFS, PCA, and the modified Zhang–Suen skeletonization method are highly susceptible to interference from overlapping hollow elliptical primitives along the connection lines and the triangular tips of the arrows, making it difficult to accurately parse the deep topological logic embedded in complex engineering drawings. The HCSA proposed in this research achieves highly efficient topology tracing and precise endpoint recognition. It accomplishes this by constructing detection circles integrated with directional masks alongside branch and extremum point-detection mechanisms. Within the textual modality, long-sequence safety-measure tickets frequently cause contextual information degradation. To resolve this specific issue, the constructed RoFormer-BiLSTM-CRF model demonstrates superior information extraction performance when compared to RoUIE, Bert, and alternative combined models. Furthermore, its inference latency is significantly lower than that of the GLM-4-FS model. Subsequently, character-level N-gram combined with TF-IDF technology and cosine similarity is adopted to align the device entities in the text and image modalities, constructing a standardized knowledge graph.

Despite the outstanding performance of the proposed method in specific scenarios, certain limitations remain. Specifically, the HCSA is primarily designed for extracting connection relationships within information flow diagrams of 500 kV substation secondary systems. Its overall applicability might decrease when evaluated on substations with different voltage levels or schematic drawings governed by alternative design specifications. Furthermore, in the text extraction and modal alignment stage, the current approach mainly utilizes traditional discriminative deep learning models and has not achieved end-to-end deep integration of the original data from different modalities.

Future research will focus on enhancing the generalization capability of the HCSA. Dynamic adjustment of the detection radius and optimization strategies for the stepping direction will be introduced, achieving adaptive tracing for paths of arbitrary curvature. Concurrently, subsequent research will also consider integrating richer heterogeneous data sources, including data from different voltage levels and different substations, and achieve cross-validation. Future research will utilize cross-modal feature fusion technologies such as graph neural networks to construct a more comprehensive knowledge graph, providing more systematic information support for the intelligent operation and maintenance and equipment information retrieval of substation secondary systems.

## 6. Conclusions

Considering the high entropy and disordered nature of multi-source heterogeneous data in substation secondary systems, an information processing method for multimodal substation data is proposed. This method utilizes information flow diagrams and safety-measure tickets to construct structured knowledge graphs and achieves the integration and visualization of physical topology connections of secondary equipment and terminal-level information. For the image modality, equipment entities are extracted by integrating YOLOv8n and OCR techniques, and the HCSA is proposed to identify information flows with an accuracy of 100%. This approach effectively addresses the challenges in recognizing equipment connectivity caused by complex line intersections and discontinuities, enabling reliable path tracing and endpoint identification of signal flow among devices in the secondary system. For the text modality, a RoFormer-BiLSTM-CRF-based information extraction model is developed. By incorporating rotary position embedding, the model effectively addresses long-range dependency issues and entity-boundary ambiguity in extended instruction sequences, enabling high-precision extraction of equipment, terminal, and operational action entities. Experimental results demonstrate that the overall performance of this model is superior to that of other combined models such as Bert. Finally, string similarity is utilized to align different modal entities, constructing a comprehensive knowledge graph for the substation secondary system, integrating both equipment connection topology and terminal operation logic.

## Figures and Tables

**Table 1:** Examples of safety-measure items.

	Item Content
Example 1	Disconnect the rear wiring terminal 4Q2D3 (101′) on the protection panel of the 5051 circuit breaker of the 500 kV WC line (500 kV Bus I bus differential protection trip 5051 circuit breaker outgoing II trip +), and wrap it properly with red insulating tape.
Example 2	Disconnect the DC power supply 2035Z of the second set of 500 kV Bus I bus differential protection panel at the front of No.1 DC distribution panel in the 500 kV protection chamber, and seal it with red insulating tape.
Example 3	Inside the terminal block of the supervisory control cabinet of the 5011 circuit breaker for the 500 kV CD line, short-circuit the current test terminal links on the CT side: TA:24 (A4171), TA:25 (B4171), TA:26 (C4171), and TA:30 (N4171) (current of the 5011 circuit breaker in the first set of 500 kV Bus I bus differential protection). After confirming that there is no current in the 5011 circuit breaker bay within the first set of 500 kV Bus I bus differential protection, open the middle links of the above terminals, and use red insulating tape to cover the terminal block of the other winding.

**Table 2:** Ontology layer of the secondary system domain in substations.

Category	Definition	Typical Examples
Entity	Device	The second set of 500 kV Bus I bus differential protection panel, the supervisory control cabinet of the 5011 circuit breaker for the 500 kV CD line.
	Loop	Inter-trip circuit, signal circuit, current circuit
	Terminal	1D2:4(GPS+), JD4(N), 3KD23
Relationship	Information Flow	Busbar PT, fault recorder signal, second group of operating circuits
	Action	Disconnect, remove, short-circuit
	Subordination	Contain, connect

**Table 3:** Performance comparison of text information extraction algorithms.

	*P*	*R*	*F*1	*T* (ms/Sample)
RoUIE	0.9480 ± 0.0039	0.9366 ± 0.0004	0.9423 ± 0.0018	54.66
GLM-4-FS	0.9502 ± 0.0158	0.9489 ± 0.0256	0.9493 ± 0.0143	302.24
Bert-BiLSTM-CRF	0.9396 ± 0.0056	0.9423 ± 0.0069	0.9409 ± 0.0048	36.61
Albert-BiLSTM-CRF	0.9484 ± 0.0075	0.9468 ± 0.0027	0.9476 ± 0.0029	33.44
Roberta-BiLSTM-CRF	0.9399 ± 0.0025	0.9464 ± 0.0061	0.9431 ± 0.0032	35.07
RoFormer-GlobalPointer	0.9375 ± 0.0054	0.9420 ± 0.0098	0.9397 ± 0.0051	26.36
ERNIE-BiLSTM-CRF	0.9074 ± 0.0046	0.9094 ± 0.0023	0.9084 ± 0.0032	35.59
RoFormer-BiLSTM-CRF	0.9506 ± 0.0031	0.9531 ± 0.0046	0.9519 ± 0.0025	36.14

**Table 4:** Ablation experiments of each module of the RoFormer-BiLSTM-CRF algorithm.

	*P*	*R*	*F*1
Bert	0.9301	0.9405	0.9353
RoFormer	0.9427	0.8878	0.9144
RoFormer-BiLSTM	0.9438	0.9470	0.9454
RoFormer-CRF	0.9277	0.9434	0.9355
RoFormer-BiLSTM-CRF	0.9527	0.9569	0.9548

**Table 5:** Typical examples of triplets.

Triplet	Example
$K_{1}$	<Supervisory control cabinet of the 5011 circuit breaker for the 500 kV CD line, 5011 circuit breaker current, the first set of 500 kV Bus I bus differential protection panel>
$K_{2}$	<500 kV WC 5381 line current transformer terminal box, short-circuit, current circuit terminal>
$K_{3}$	<Inter-trip circuit terminal, contain, 3KD20 (SL41)>

## Data Availability

The original contributions presented in this study are included in the article. Further inquiries can be directed to the corresponding author.
